# Supplementary material for: Changes in Physical Activity, Sitting and Sleep across the COVID-19 National Lockdown Period in Scotland
Source: Int J Environ Res Public Health. 2020 Dec 14;17(24):9362. doi: 10.3390/ijerph17249362 (PMC7765067; doi:10.3390/ijerph17249362)
Supplement: Supplementary file 1 [file ijerph-17-09362-s001.pdf]

Supplementary tables

Table S1. Sleep recommendations' categorization.

	Insufficient	Appropriate (Low)	Recommended	Appropriate (High)	Too Much
18–25 year olds	< 6 h	6 h	7 to 9 h	10 to 11 h	≥ 12 h
26–64 year olds	< 6 h	6 h	7 to 9 h	10 h	≥ 11 h
+65 year olds	< 5 h	5 to 6 h	7 to 8 h	9 h	≥ 10 h

Table S2. Differences between included and drop out participants.

	Included			Drop Out [a]			
	N	Mean [b]	SD	N	Mean [b]	SD	p
**T1-T2**							
Sitting per day (min)	2297	396.80	189.12	255	397.91	178.12	0.929
MVPA per week (min)	2358	358.17	522.30	168	518.35	679.34	<0.001
Walk per week (min)	2363	476.10	523.47	332	496.36	507.06	0.508
Sleep per night (h)	2458	6.60	1.22	758	6.58	1.26	0.802
**T2-T3**							
Sitting per day (min)	1495	420.44	207.48	845	439.77	216.28	0.033
MVPA per week (min)	1548	406.98	574.96	873	461.78	682.77	0.045
Walk per week (min)	1557	427.93	404.56	855	404.39	452.01	0.190
Sleep per night (h)	1608	6.88	1.51	865	6.87	1.62	0.868

[a] Drop out from T1 to T2 or T2 to T3; [b] Mean indicates mean at T1 (for T1-T2) and mean at T2 (for T2-T3).

Table S3. Number of participants in each movement behavior category at T1 (rows) and T2 (columns).

			MVPA (min/week)				
	<30	30 to <60	60 to <150	150 to <300	300+	Total	Positive changers (%)
<30	265	14	72	61	129	541	51.02
30 to <60	9	8	11	12	5	45	62.22
60 to <150	81	10	106	82	117	396	49.74
150 to <300	64	7	60	127	193	451	42.79
+300	94	16	95	120	600	925	NA
Total	513	55	344	402	1044	2358	
			**Walk (min/week)**				
	<30	30 to <60	60 to <150	150 to <300	300+	Total	Positive changers (%)
<30	45	7	27	23	52	154	70.78
30 to <60	10	5	21	16	16	68	77.94
60 to <150	36	9	78	60	156	339	63.72
150 to <300	28	16	79	122	187	432	43.3
300+	106	27	162	177	898	1370	NA
Total	225	64	367	398	1309	2363	
			**Sitting (h/day)**				
	<4	4 to <6	6 to <8	8+		Total	Positive changers (%)
<4	188	120	61	36		405	NA
4 to <6	86	232	118	120		556	15.47
6 to <8	46	96	160	184		486	29.22
8+	35	76	101	638		850	24.94
Total	355	524	440	978		2297	
			**Sleep (h/night)**				
	Insufficient	Appropriate (low)	Recommended	Appropriate (high)	Too much	Total	Positive changers (%)
Insufficient	157	59	122	3	3	344	53.49
Appropriate (low)	128	251	385	17	4	785	49.04
Recommended	122	199	924	48	6	1299	NA
Appropriate (high)	0	1	6	9	3	19	31.58
Too much	0	0	2	1	1	4	50.00
Total	407	509	1439	78	17	2451	

Footnote: a positive change is illustrated in green, a negative change is illustrated in orange and no change is illustrated in yellow.

Table S4. Number of participants in each movement behavior category at T2 (rows) and T3 (columns).

	**MVPA (min/week)**						Positive changers (%)
	**<30**	**30 to <60**	**60 to <150**	**150 to <300**	300+	Total	
<30	198	12	48	25	66	349	43.27
30 to <60	13	1	12	6	7	39	64.10
60 to <150	50	8	49	41	68	216	50.46
150 to <300	36	6	40	81	104	267	38.95
300+	58	3	44	87	485	677	NA
Total	355	30	193	240	730	1548	
	**Walk (min/week)**						
	<30	30 to <60	60 to <150	150 to <300	300+	Total	Positive changers (%)
<30	37	8	35	21	41	142	73.94
30 to <60	5	1	13	7	10	36	83.33
60 to <150	26	25	58	61	59	229	52.40
150 to <300	12	15	38	81	114	260	43.85
300+	31	41	37	120	661	890	NA
Total	111	90	181	290	885	1557	
	**Sitting (h/day)**						
	<4	4 to <6	6 to <8	8+		Total	Positive changers (%)
<4	126	74	22	10		232	NA
4 to <6	104	151	67	49		371	28.03
6 to <8	44	85	82	65		276	46.74
8+	40	52	120	404		616	34.42
Total	314	362	291	528		1495	
	**Sleep**						
	Insufficient	Appropriate (low)	Recommended	Appropriate (high)	Too much		Positive changers (%)
Insufficient	131	93	27	0	0	251	47.81
Appropriate (low)	44	186	111	2	0	343	32.36
Recommended	53	178	700	12	2	945	NA
Appropriate (high)	1	5	35	13	1	55	63.64
Too much	2	1	4	2	0	9	77.78
Total	231	462	877	29	3	1603	

Footnote: a positive change is illustrated in green, a negative change is illustrated in orange and no change is illustrated in yellow.
